# European Bat Lyssaviruses, the Netherlands

**DOI:** 10.3201/eid1112.041200

**Published:** 2005-12

**Authors:** Wim H.M. Van der Poel, Reina Van der Heide, Elisabeth R.A.M. Verstraten, Katsuhisa Takumi, Peter H.C. Lina, Johannes A. Kramps

**Affiliations:** * National Institute for Public Health and the Environment, Bilthoven, the Netherlands; † Central Institute for Animal Disease Control, Lelystad, the Netherlands; ‡ National Museum of Natural History, "Naturalis", Leiden, the Netherlands

**Keywords:** EBLV, lyssavirus, the Netherlands, bat, Eptesicus serotinus, Myotis dasycneme, Europe, research

## Abstract

To study European bat lyssavirus (EBLV) in bat reservoirs in the Netherlands, native bats have been tested for rabies since 1984. For all collected bats, data including species, age, sex, and date and location found were recorded. A total of 1,219 serotine bats, *Eptesicus serotinus*, were tested, and 251 (21%) were positive for lyssavirus antigen. Five (4%) of 129 specimens from the pond bat, *Myotis dasycneme*, were positive. Recently detected EBLV RNA segments encoding the nucleoprotein were sequenced and analyzed phylogenetically (45 specimens). All recent serotine bat specimens clustered with genotype 5 (EBLV1) sequences, and homologies within subgenotypes EBLV1a and EBLV1b were 99.0%–100% and 99.2%–100%, respectively. Our findings indicate that EBLVs of genotype 5 are endemic in the serotine bat in the Netherlands. Since EBLVs can cause fatal infections in humans, all serotine and pond bats involved in contact incidents should be tested to determine whether the victim was exposed to EBLVs.

## Abstract 2

Genotype 5 lyssaviruses are endemic in the Netherlands, and can cause fatal infections in humans.

European bat lyssaviruses (EBLVs) belong to the *Lyssavirus* genus of the *Rhabdoviridae* family and form a group of negative single-stranded RNA viruses with an almost worldwide distribution. The genus *Lyssavirus* can be divided into 7 genotypes, including EBLV1 as genotype 5 and EBLV2 as genotype 6 (*1*). EBLVs have been demonstrated in several bat species, and more bat species may be susceptible. The bite of an EBLV-infected bat may cause fatal encephalitis in humans; 4 fatal human cases have been reported. Three of the 4 viruses were typed genetically to be definitely EBLV (*2*). The most recent case was a 56-year-old bat worker in Scotland who was thought to have been bitten on his hand by a Daubenton's bat (*3*). Therefore, bat rabies is considered a public health threat in countries where these viruses are endemic in bats. In the Netherlands, genotype 1 lyssaviruses were eradicated in the early 1990s, but EBLVs are endemic in several bat species; the serotine bat, *Eptesicus serotinus*, is considered the main reservoir of rabies in this country. If possible, bats involved in contact incidents are sent to the Central Institute for Animal Disease Control (CIDC), Lelystad, to be tested for lyssavirus. Rabies diagnosis is always performed within 24 hours and if necessary, rabies postexposure prophylaxis is administered to the patient according to World Health Organization recommendations.

To provide a picture of rabies incidence and distribution in native bat species in the Netherlands, data for bats tested for lyssavirus antigen at CIDC-Lelystad were collected and analyzed. To characterize the circulating EBLVs, reverse transcription–polymerase chain reaction (RT-PCR) amplification products of EBLV RNA were sequenced and analyzed phylogenetically.

## Materials and Methods

### Bat Specimens and Bat Data

In the Netherlands, surveillance of lyssaviruses in bats is ongoing. In 1984, it became mandatory to submit bats caught by animals or bats that were unable to fly to the CIDC-Lelystad Department of Virology for lyssavirus antigen testing. After 1987, surveillance began nationwide; since 1994, mainly bats involved in contact incidents and suspected of rabies infection have been submitted for testing. As part of this surveillance, from 1984 to 2003, brain tissue samples were collected from all submitted bats. Each year, >100 of these animals were tested for lyssavirus antigen. The locations where the bats were found were plotted by using 5 × 5 km grid allocations. In addition to location and date found, species, sex, and age were determined and recorded (by P.H.C.L.).

The number of EBLV-positive bats was described by the binomial distribution prevalence that was specific to each sex-age group. This distribution produced a sequence of nested binomial models to test the effect of adding an extra group-specific prevalence to a simpler model by the likelihood ratio test. If the resulting deviance was greater than the 95th percentile of the chi-square distribution with 1 degree of freedom, the prevalence of the sex-age group was considered to be significantly different from the others.

### Detection of Lyssavirus Antigen

Detection of lyssavirus antigen was performed by standard fluorescent antibody test (FAT) as described (*4*), with modifications, using polyclonal fluorescein isothiocyanate–labeled rabbit anti-rabies nucleocapsid immunoglobulin G (IgG) (Diagnostics Pasteur, Marnes-la-Coquette, France). Positive controls, brain tissue smears from mice infected with genotypes 1 and 5 field virus, were incorporated in each test run. Duplicate smears were carefully and completely checked for fluorescence. From 1997 to 2003, all test results that were positive by FAT (n = 45) were confirmed by RT-PCR and sequenced.

### RT-PCR Analyses

To amplify EBLV-specific RNA, brain tissue samples (3 mm^3^) were put in 0.5 mL RNA extraction buffer. The RNA extraction was performed by using TRIzol (Invitrogen Life Technologies, Merelbeke, Belgium) according the manufacturer's protocol. TRIzol was added to the brain tissue sample to a total volume of 1.0 mL. RT-PCR amplification was performed as described (*5*). Primer selection and Southern blot hybridizations of RT-PCR products were performed as described (*6*). To characterize the EBLVs, RT-PCR amplification products of all FAT-positive samples collected from 1997 to 2003 were confirmed by RT-PCR and sequenced later.

### EBLV RNA Sequence Analyses

Direct sequencing of the RT-PCR-amplified products of a 566-nucleotide (nt) fragment coding for the amino terminus of the nucleoprotein of EBLV and analyses of the nucleotide sequences were performed as described (*7*). This part of the genome was used to enable a comparison with sequences described by other lyssavirus researchers in Europe (*7*). PCR fragments were purified by QIAquick Purification Kit (Qiagen, Hilden, Germany) and then sequenced directly on both strands. Sequencing was performed on a Biosystems (ABI) 3700 DNA automated sequencer (Perkin Elmer-Applied Biosystems, Foster City, CA, USA) by using fluorescent dye–labeled dideoxynucleo terminators (BigDye Terminator Cycle Sequencing Ready Reaction, Perkin Elmer-Applied Biosystems, Warrington, UK). Nucleotide sequences were edited by using Seq Ed (V1.03, Applied Biosystems), and aligned using Bionumerics (V2.5) (Applied Maths, Kortrijk, Belgium). To compare detected and historic sequences, phylogenetic trees were created based on cluster analyses and global alignment similarities of 396 nucleotide fragments of the N-gene encoding region (position in the genome nucleotide 46–441, numbered according to the CVS strain, GenBank accession no. D42112). The confidence values of the internal nodes were calculated by performing 100 bootstrap analyses (Bionumerics V2.5).

To allow the geographic relationship between homologous strains to be studied, sequences showing a high sequence homology were grouped by "cluster." All sequence "cluster" numbers were positioned on the country map to determine if sequences with high homogeneity originated from the same region.

## Results

### Detection of Lyssavirus Antigen

**Figure 1:**
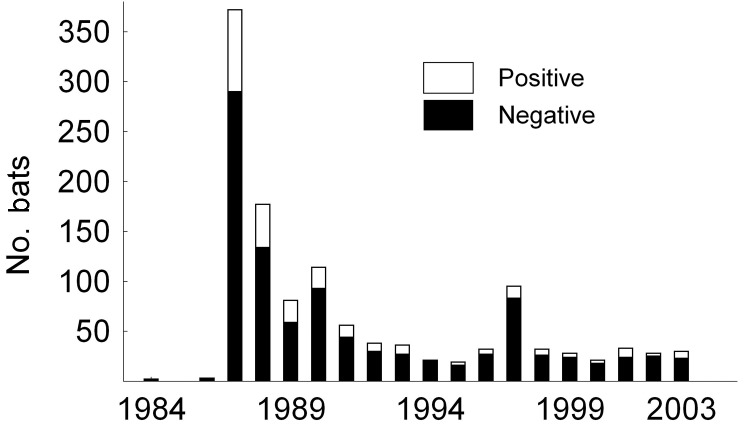
Number of submitted and lyssavirus antigen–positive samples from serotine bats, Eptesicus serotinus, collected in the Netherlands during the survey (1984–2003).

From 1984 to 2003, bats of 1 vagrant and 11 native species (Table 1) were tested for lyssavirus antigen. Lyssavirus was detected in 2 species only, the serotine bat and the pond bat, *Myotis dasycneme*. A total of 1,219 serotine bats and 129 pond bats were tested for lyssavirus antigen; 251 serotine bats and 5 pond bats were positive, which results in 21% and 4% prevalence, respectively. In the most common native bat species, the pipistrelle, *Pipistrellus pipistrellus*, EBLV was never detected (1,837 specimens tested). Approximately one third (32%) of all bats submitted for lyssavirus antigen testing were serotine bats (Table 1,Figure 1).

**Table 1:** Investigated bats, the Netherlands, 1984–2003

Bat species	No. specimens tested	Lyssavirus antigen positive (%)
*Eptesicus serotinus*	1,219	251 (20.6)
*E. nilssonii*	1	0
*Myotis mystacinus*	18	0
*M. nattereri*	9	0
*M. daubentonii*	111	0
*M. dasycneme*	129	5 (3.9)
*Pipistrellus pipistrellus*	1,837	0
*P. nathusii*	256	0
*Nyctalus noctula*	61	0
*N. leisleri*	3	0
*Plecotus auritus*	214	0
*Vespertilio murinus*	6	0
Undetermined	9	0
Total	3,873	256

### EBLV RT-PCR and RNA Sequence Analyses

**Figure 2:**
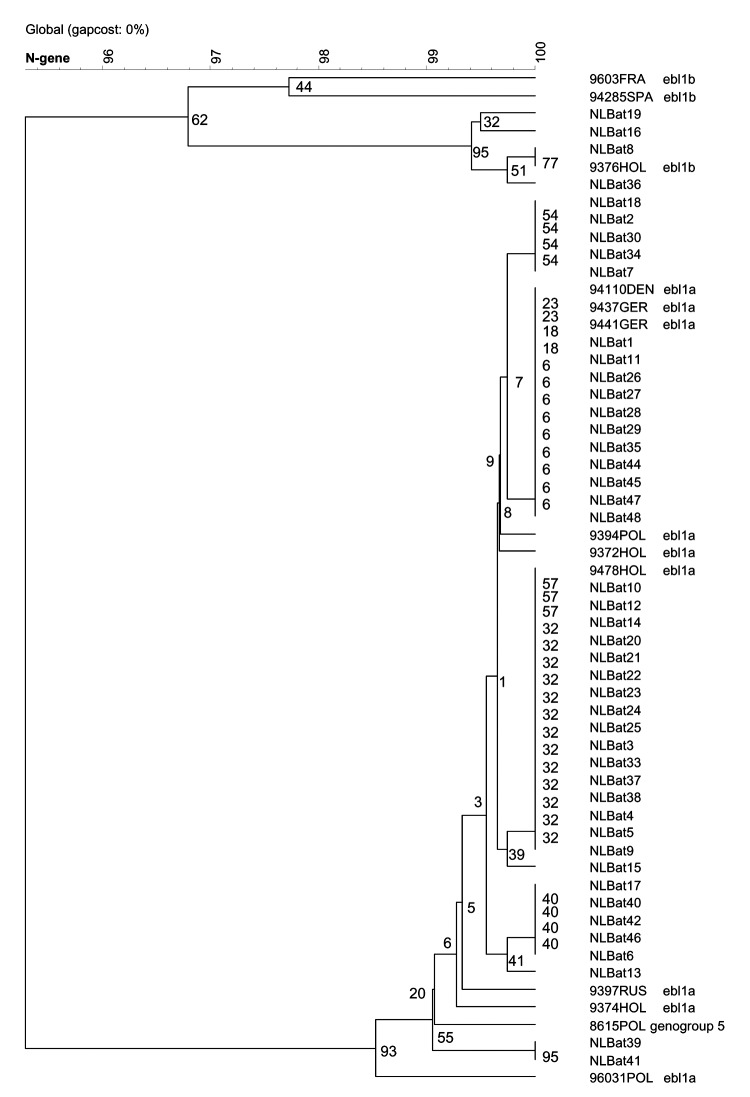
Phylogenetic tree of European bat lyssavirus (EBLV1) sequences detected in serotine bats in the Netherlands, 1997–2003, and historic EBLV sequences detected in bats in Europe. Tree calculated based on cluster analyses and global alignment similarities of 396 nucleotide fragments of the N-gene encoding region (position in the genome nucleotide 46–441, numbered according to the CVS strain, GenBank accession no. D42112). The confidence values of the internal nodes were calculated by performing 100 bootstrap analyses.

**Figure 3:**
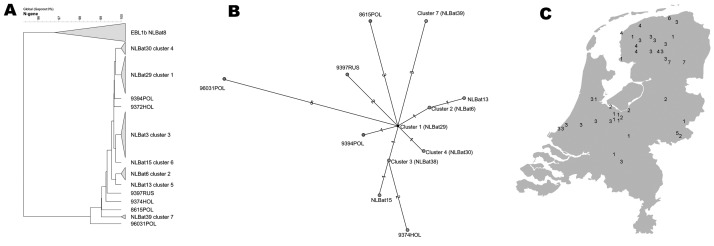
A) Phylogenetic tree of European bat lyssavirus 1 (EBLV1) sequences detected in serotine bats in the Netherlands, 1997–2003, and some historic sequences detected in bats in Europe. Analysis performed with maximum parsimony of representative DNA sequences of different EBLV1 sequences. B) Relationships between 7 different serotine bat EBLV1a sequence lineages (numbered "clusters" 1 to 7): maximum parsimony unrooted tree of representative EBLV1 sequences detected in serotine bats in the Netherlands and several historic sequences detected in other European countries (color coded country). C) Geographic distribution of the 7 different serotine bat EBLV1 lineages (numbered "clusters" 1 to 7) from 41 recently detected serotine bat isolates in the Netherlands.

All FAT-positive bat brain tissue samples collected from 1997 to 2003 tested positive by PCR. Direct sequencing of the RT-PCR–amplified products of a 566-nt coding region of the amino terminus of the nucleoprotein resulted in homologies within subgenotypes EBLV1a (41 specimens) and EBLV1b (4 specimens) of 99.0%–100% and 99.2%–100%, respectively. Homologies with older EBLV bat isolates from the Netherlands were also within these ranges. Phylogenetic analyses did not show a significant change or shift in EBLV nucleoprotein encoding sequences over the years (Figures 2 and 3).

### Geographic Distribution, Age, and Sex

**Figure 4:**
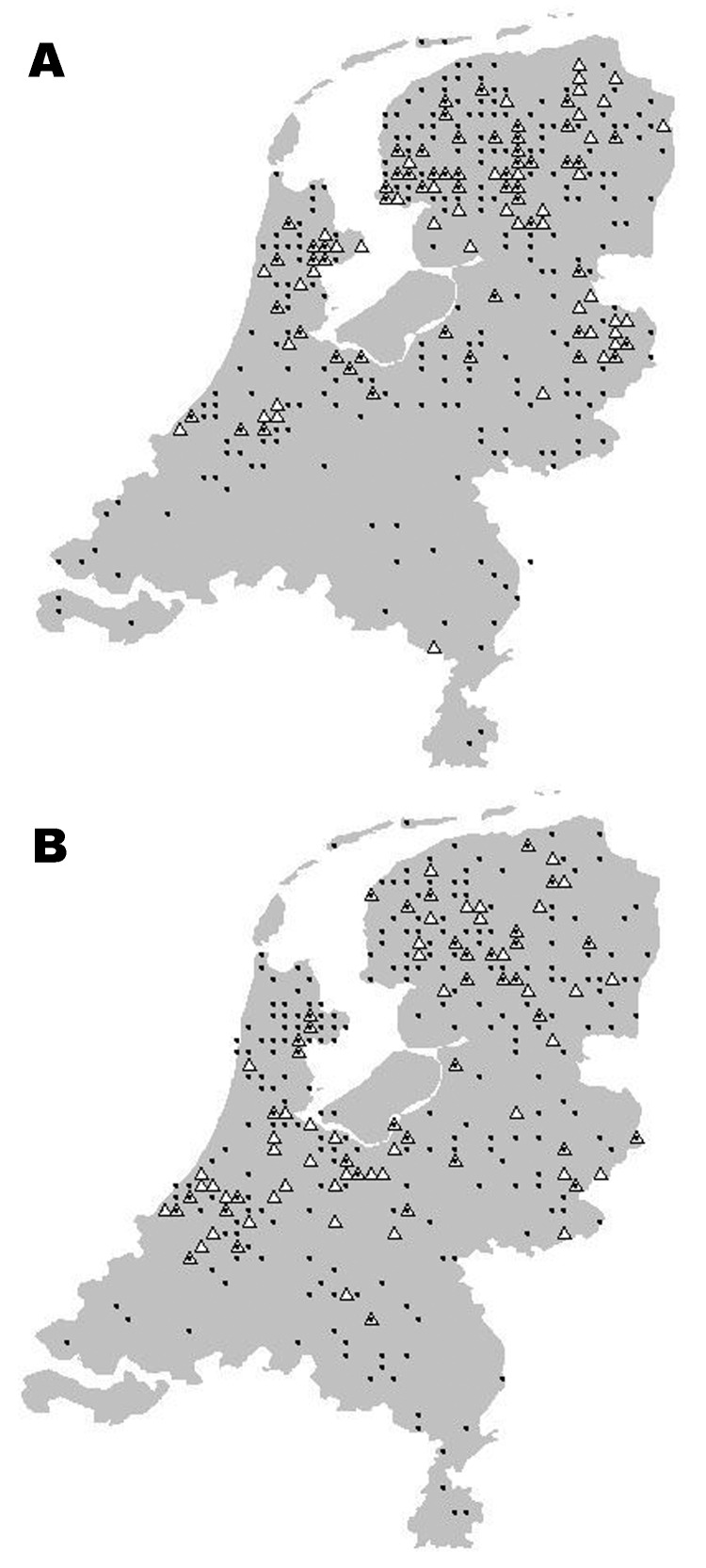
Location of serotine bat, Eptesicus serotinus, with positive (triangles) and negative (dots) test results for European bat lyssaviruses, the Netherlands; A)1984–1989; B)1990–2003.

**Figure 5:**
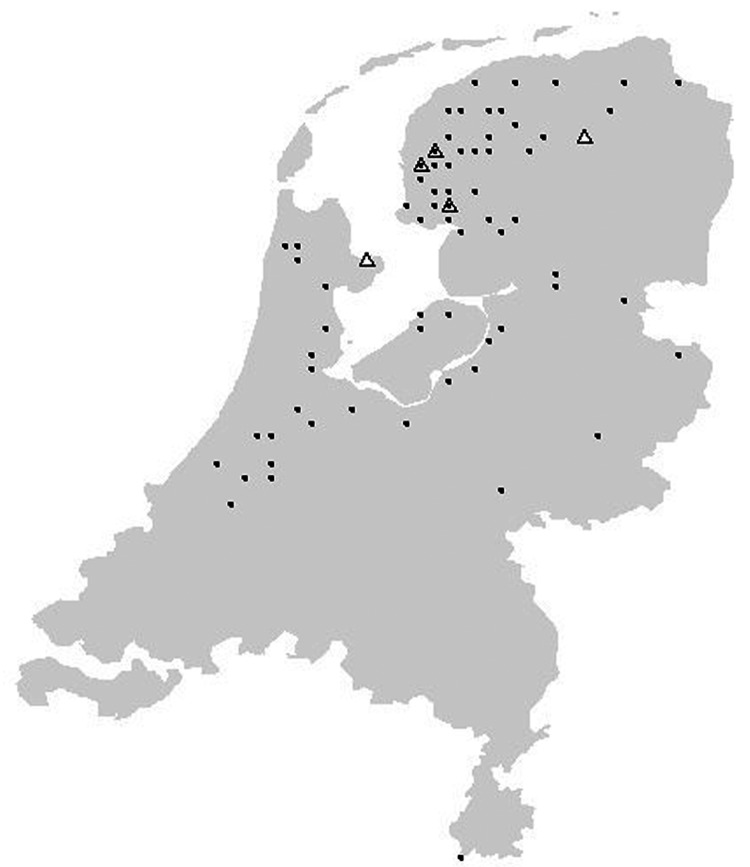
Location of pond bat, Myotis dasycneme, with positive (triangles) and negative (dots) test results for European bat lyssaviruses, the Netherlands, 1984–2003.

The geographic origin of tested and EBLV-positive serotine and pond bats are depicted in Figures 4 and 5, respectively. The numbers of tested and EBLV-positive serotine bats per year are shown in Figure 1. High homology sequences detected in serotine bats did not show a clustering per year (Figure 2), but EBLV sequences that showed a high degree of homology seemed to have a geographic relationship for at least 2 lineages (clusters 2 and 4, Figure 3). Regarding age and sex, a significantly higher number of EBLV-infected serotine bats were found in the group of adult females, 25% EBLV positives (Table 2).

**Table 2:** European bat lyssavirus–positive serotine bats by age and sex

Sex/age group	Examined	Positive	Prevalence (%)
Male juvenile	22	6	27
Female juvenile	35	4	11
Male adult	684	132	19
Female adult*	438	108	25
Subtotal	1,178	250	21
Sex and age unknown	8	0	
Adult/unknown sex	24	1	4
Juvenile/unknown sex	8	0	
Total	1,219	251	21

*Prevalence for this group was significantly higher than for the rest based on likelihood ratio test.

## Discussion

From 1984 to 2003, the serotine bat appeared to be the main wildlife reservoir of EBLVs in the Netherlands, as in several other European countries (*7*). European bat lyssavirus incidence in serotine bats in the Netherlands was 21%. A much lower incidence of 4% was found in the pond bat. In the Netherlands, females of both the serotine bat and the pond bat usually dwell as maternity colonies in the summer; single males or male groups dwell in walls of houses and other buildings and occasionally in suitably quiet spaces like church lofts.

These practices may bring humans and pets in contact with diseased bats. Among pets, cats are the most common predators of building-dwelling bats. Most of the bats submitted for testing were prey of cats, many of the bats had been in direct contact with humans, and only a few were involved in biting incidents. Most of these bats were assumed to be diseased or exhausted, and therefore, the observed EBLV incidences in both bat species almost certainly are an overestimation of the incidence in healthy bat populations in the Netherlands. EBLV-positive cats were never identified, so the risk of infection seems to be very low for cats involved in bat contact.

The difference in the overall prevalence between the serotine bat and the pond bat is difficult to explain. The serotine bat and the pond bat are not related species. Differences in behavior or sensitivity to EBLV infection or disease may be underlying causes for the observed differences in EBLV prevalence in the 2 species. The pathogenicity of EBLVs in natural hosts has not been extensively studied because bats are legally protected in most European countries (European Commission Directive 92/43/EEC on the Conservation of Natural Habitats and of Wild Fauna and Flora, 1992). Some European countries have even more strict regulations than those described in the European directive. Due to strict regulations on wildlife conservation in some countries, the study of EBLV infections in bats is hampered. Therefore, whether EBLVs normally cause fatal infections or induce an asymptomatic infection and virus persistence in individual bats is not known (*8*). Another possibility is that EBLVs induce infection with a long incubation period (i.e., months, years), because this can also lead to long-term maintenance of the virus within a specific species. Studies to elucidate this issue have not shown exclusive results (*6*).

Apart from the species, data including age, sex, and dates and locations the bats were found were also documented; these data enable us to show a clear picture of the geographic distribution of EBLVs in different bat species in the Netherlands (Figures 4A and B and Figure 5) for the first time. The serotine bat is relatively common and can be found throughout the country. The species is especially numerous in the northwest. In the south, the species is present almost everywhere but in rather low numbers. The pond bat is fairly common in the north and west. Both serotine bats and pond bats submitted for EBLV testing were concentrated more in the northern and the middle part of the country. This finding indicates the summer distribution and population density of both species in the Netherlands (*9*). During winter, the serotine bat hibernates individually or in small groups in cavity walls of buildings usually not far from its summer roosts. The pond bat migrates to subterranean winter roosts which may be 20–300 km from its summer roosts. Hibernation of the pond bat in cavity walls of buildings has not yet been observed. The higher number (25%) of EBLV infections observed in adult female serotine bats is not surprising because adult females of this species live close together during their maternity period in the summer.

Forty-five FAT-positive bat brain tissue samples selected for EBLV sequence analysis tested positive by PCR. For these samples, the EBLV RT-PCR assay proved to be at least as sensitive as the FAT test system that is used for EBLV surveillance. Sequencing the RT-PCR-amplified products of the nucleoprotein-encoding region and subsequent sequence analyses resulted in exclusively EBLV1 subgenotype lineages. EBLV1a isolates showed a 99.0%–100% homology, whereas EBLV1b isolates in the Netherlands showed a 99.2%–100% homology. Homologies with older EBLV isolates from the Netherlands were also within these ranges (Figures 2 and 3).

Sequences from serotine bats with high homology for at least 2 EBLV1a lineages (clusters 2 and 4, Figure 3) originated from a defined geographic region in the Netherlands. This observation suggests that transmission of EBLVs in serotine bats over long distances within the country does not seem to play a major role in EBLV epidemiology. However, Davis et al. (*10*) recently suggested that transmission viral traffic may be established among bats in northern Europe because high homology strains were found across this entire region. For the EBLV1b lineages, the possibility of geographic clustering of high homology sequences could not be determined because of the relatively low number of analyzed sequences.

EBLV sequences detected in native bats in the Netherlands showed little divergence and did not indicate an emergence of new EBLV strains, but this study confirms that the serotine bat is an EBLV1 reservoir. Since at least 4 fatal EBLV infections have been reported in humans in Europe in the last decade, the public health hazard of bat rabies in Europe should not be underestimated. Any contact with bats in Europe must be considered possible exposure, and biting incidents should be treated immediately with rabies postexposure prophylaxis. If possible, the involved bat should always be kept for lyssavirus testing. To prevent rabies transmission from bats, all bat handlers should be informed of the risks of rabies exposure and advised to be vaccinated. The continuing prevalence of EBLVs in serotine bats and pond bats in Europe and the risk of a fatal infection in humans should compel European countries to work together on bat lyssavirus surveillance.
